# A pilot study of a hospital-based injury surveillance system in a secondary level district hospital in India: lessons learnt and way ahead

**DOI:** 10.1186/s40621-016-0090-7

**Published:** 2016-11-03

**Authors:** P. V. M. Lakshmi, Jaya Prasad Tripathy, Nalinikanta Tripathy, Sunita Singh, Deepak Bhatia, Jagnoor Jagnoor, Rajesh Kumar

**Affiliations:** 1 Department of Community Medicine & School of Public Health, Post Graduate Institute of Medical Education & Research, Chandigarh, India; 2 International Union Against Tuberculosis and Lung Disease, The Union South East Asia Office, New Delhi, India; 3 Kalinga Institute of Medical Sciences, Bhubaneswar, Odisha India; 4 Integrated Disease Surveillance Project, Punjab, India; 5 Injury Division, The George Institute for Global Health, University of Sydney, Sydney, Australia

**Keywords:** Injury surveillance, Hospital-based, India

## Abstract

**Background:**

Reliable epidemiological information on injury burden and pattern is essential to formulate effective injury control and prevention strategies. Injury surveillance systems are globally gaining ground as a tool for collecting such systematic data on injuries, but less so in low and middle income countries. This study describes the experience of setting up a District Level Hospital-Based Injury Surveillance System in India and the pattern of injuries encountered therein.

**Methods:**

A prospective study was conducted during Jan-Dec 2012 at the emergency department of a District Hospital in Fatehgarh Sahib in a North Indian state of Punjab. A comprehensive injury proforma was devised to record information on all injury cases reporting to the hospital. Emergency Medical Officers were trained to record data.

**Results:**

A total of 649 injuries were reported in 2012. The surveilance system used the existing resources at the hospital to collect data without the need for additional manpower, equipments etc. About 78 % of injuries reported were unintentional in nature. More than half (52.9 %) of the patients had injuries due to Road Traffic Crashes. Head (29.7 %) was the most common site of injury. Incised injury (50.2 %) was the most common type of injury and most of the injuries occurred while travelling (61.8 %).

**Conclusion:**

Developing better and sustainable systems of routine injury surveillance or trauma registries is essential to generate reliable information for formulating effective intervention policies.

## Background

Injuries rank among the leading causes of morbidity and mortality in the world with a steady increase in developing countries like India. About 5.8 million people die each year as a result of injuries. This accounts for 10 % of the world’s deaths, 32 % more than the number of fatalities that result from malaria, tuberculosis and HIV/AIDS combined (Injuries and violence 2010). Approximately 90 % of the global injury-related deaths occur in low and middle income countries, like India (Injuries and violence 2010). India is a LMIC with more than one billion people, and one of the fastest growing economies in the world. The economic growth has also meant a rapidly increasing number of vehicles sold every year (around six million) and one of the highest reported mortality rates from road traffic injuries in the world (Garg & Hyder 2006). Traffic fatalities have increased by about 5 % per year from 1980 to 2000, and since then have increased by about 8 % per year (Mohan et al. 2009). The burden of traumatic injuries in India is certainly high, but remains ill-defined and poorly quantified.

For example, there are currently no comprehensive studies, to our knowledge, that document the burden of non-fatal injuries in India. Systematic and scientific efforts in injury prevention and control have gained little momentum, largely due to lack of data on the epidemiology of injuries. Data on injuries is essential to prioritize evidence based safety strategies and prevention efforts. Reliable estimates on injury burden and pattern will also aid in organization and delivery of acute trauma care. Thus, there is greater emphasis on the need to generate reliable and consistent information on the pattern and distribution of injuries so as to design effective prevention strategies. Consequently, injury surveillance systems are widely gaining ground as a tool for collecting such systematic data on injuries. Although they are well-developed in high resource settings, they are almost non-existent in resource poor Low and Middle income countries (LMICs). This study describes the experience of setting up an Injury Surveillance System in a District Level Hospital in India and the pattern of injuries encountered therein. It also aimed at exploring the feasibility of a secondary level hospital-based surveillance system in a resource poor setting with high injury burden and also to understand its challenges and constraints.

## Methods

### Study setting

The study was carried out at the emergency department of District Hospital, Fatehgarh Sahib, Punjab. Fatehgarh Sahib is a district in Punjab, a state in North India with a population of approximately 600 000. The district has a reasonably good spread of health services. It has 1 District Hospital, 1 sub-divisional hospital, 2 primary health centres (PHCs), 3 community health centres (CHCs) and 11 Mini-PHCs besides other rural hospitals and dispensaries. An injury surveillance project was started at the Emergency Department (ED) of the District Hospital after taking due permission from the State Surveillance Unit and the concerned district health officials. The District Hospital provides specialized secondary care services catering to a population of around 0.6 million. It is staffed by specialists from the departments of medicine, surgery, pediatrics, orthopedics, anesthesia, dermatology, psychiatry, ophthalmology, pathology, dental and 5 medical officers. A comprehensive injury proforma was developed based on WHO Injury Surveillance guidelines (Holder et al. 2001). The questionnaire had three major sections: patient demographics, details of the injury and events after injury. The details of the injury, amongst others, included the intent, mechanism, nature and site of injury, activity that the patient was involved in when the injury occurred, place of injury and alcohol or substance use. (Attached as annexure) The Emergency Medical Officers were trained to fill the forms. Data were collected for all patients admitted to the emergency department during the period January-December 2012. An electronic database was developed on Epi Info. The data collected was entered into the database and exported to SPSS 16 for analysis (SPSS Inc 2007). Checks were built into the Epi-Info data collection tool to ensure data quality. Random check of the data (10 %) was also done.

### Description of the injury surveillance system

Injury Surveillance System was initiated at the District Hospital in Fatehgarh Sahib following discussions with the Civil Surgeon, medical officers and other key officials. Medical Officers in the hospital were trained to collect information from the patients who visited the emergency department with an injury on a structured proforma. Upon arrival of a patient in the emergency department, routine admission procedures and management as per the treatment protocols were followed. No changes were made to the usual care of the patient. The trained emergency medical officer after stabilizing the patient collected information on socio-demographics and injury history from the attendant or the patient, if able, after informed consent. The intent, mechanism, site and nature of injury was based on the assessment of the Emergency Medical Officer (EMO) filling the form. The data were entered on a weekly basis at the Surveillance Unit at School of Public Health, Post Graduate Institute of Medical Education and Research, (PGIMER) Chandigarh, India, which is a premier tertiary care medical institute. The Injury Surveillance System was coordinated by the Surveillance Unit at School of Public Health, PGIMER led by the Head of the Department and the Faculty of Epidemiology at the School of Public Health. Other members of the unit were Senior and Junior Residents of Community Medicine, Demonstrator in Epidemiology, Public Health Nurse and a Data Entry Operator.

The data quality was ensured by the Public Health Nurse, Senior Resident and the faculty visiting the hospital weekly by checking the data of the form with the data available in the registers of the emergency department on the numbers and whether all the columns were properly filled or not. Data were entered by a Data Entry Operator at the Surveillance Unit at School of Public Health, PGIMER, Chandigarh.

### Data analysis

Descriptive statistics such as percentages have been used to summarize categorical variables. Chi-square test was done to study the association between different types of injuries and demographic variables such as age group and sex.

## Results

Out of a total of 1548 patients admitted to the emergency department in the year 2012, 649 (42 %) cases of injuries were reported through the surveillance system. A total of 628 medico-legal cases (MLCs) were registered in the same year. As most of the medico legal cases comprises of injuries, the number recorded in the surveillance system should not be less than the reported medico legal cases. (Medico-Legal Case are defined as a case of injury or ailment and like in which investigation by the law-enforcing agencies is essential to fix the responsibility regarding the causation of the said injury or ailment) (Wikipedia contributors). The surveilance system used the existing resources at the hospital to collect data without the need for additional manpower, equipments etc.

### Basic socio-demographic profile of patients

A total of 649 patients were reported by the injury surveillance system during the study period. Around 40 % (254) of patients belonged to the age group 25–44 followed by 45–64 with 21.7 % (140). More males (80 %, 517) reported injuries as compared to females (20 %, 130) Table 1.

**Table 1:** Socio-demographic profile and characteristics of injuries among patients attending the emergency of a Secondary Level Hospital in District Fatehgarh Sahib, Punjab, India, 2012 (*N* = 649)

Characteristics	Number (%)
Sex	
Male	517 (80)
Female	130 (20)
Not recorded	02 (0)
Residence	
Urban	302 (47)
Rural	312 (48)
Not recorded	35 (5)
Age group	
0–14	53 (8.2)
15–24	176 (27.1)
25–44	254 (39.1)
45–64	140 (21.6)
> 64	22 (3.4)
Not recorded	04 (0.6)
Intent of injury	
Unintentional	493 (78.1)
Intentional	122 (19.4)
Self-harm	12 (1.9)
Others	4 (0.6)
Not recorded	18 (2.8)
Site of injury^c^	
Head	190 (29.3)
Face	142 (21.9)
Upper limb	140 (21.6)
Lower limb	102 (15.7)
Chest	26 (4.0)
Spinal	16 (2.5)
Neck	12 (1.8)
Abdomen	10 (1.6)
Bone	1 (0.1)
Not recorded	64 (10)
Nature of Injury^c^	
Incised wound	326 (50)
Fracture/Dislocation	113 (17)
Abrasion	112 (17)
Contusion	44 (7)
Concussion	36 (6)
Laceration	35 (5)
Burn	12 (2)
Others^a^	11 (2)
Not recorded	36 (6)
Activity during injury	
Travelling	330 (51)
Working	150 (23)
Sports	18 (3)
Education	18 (3)
Others	23 (3)
Not recorded	110 (17)
Mechanism of injury	
Road Traffic Crash	338 (52)
Quarrel	110 (17)
Fall	80 (12)
Stab cut	36 (6)
Other blunt force	22 (3)
Animal bite	20 (3)
Fire	7 (1)
Poisoning	5 (1)
Others^b^	19 (3)
Not recorded	12 (2)
Outcome of injury	
Treated and discharged	336 (51.8)
Admitted to indoor wards	171 (26.3)
Referred to higher centres	109 (16.8)
Death	6 (0.9)
LAMA	6 (0.9)
Not recorded	21(3.2)

^a^includes internal organ injury, nose bleeding, crush injury and foreign body

^b^includes unknown, drowning and gun shot; LAMA stands for Left Against Medical Advice

^c^multiple responses

#### Profile of injuries

About 78 % (493) of injuries reported were unintentional followed by intentional injuries in 19 % (122) of cases. More than half (52.9 %, 338) of the patients had injuries due to road traffic crashes, head (29.7 %, 190) was the most common site of injury. Among the nature of injuries, incised wound (clean cut wound usually by a sharp instrument) (50.2 %) was the most common. (See Table 1) Around 90 % (259) of crash victims did not wear a helmet or seatbelt. Among the cases of road traffic crashes, most of them were travelling on a two-wheeler (51.8 %, 171), followed by car/auto (17.5 %, 57) and pedestrians (14.4 %, 47) (data not shown).

**Fig. 1:**
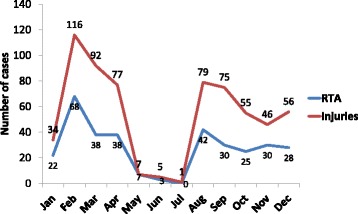
Seasonal variation of injuries and road traffic crashes as reported by the injury surveillance system at a Secondary Level Hospital in District Fatehgarh Sahib, Punjab, India, 2012

Table 2 provides the cross tabulations between the intent and the nature of injury. Nearly half (50.1 %, 247) of unintentional injuries were cut/open wounds followed by fractures (10.8 %, 53). Cut/open wounds accounted 66.7 %, 8) of self-inflicted injuries and (42.6 %, 52) of intentional injuries. (See Table 2) Table 3 gives the distribution of intent of injury by age group and sex. Both intentional and unintentional injuries were common among males in the age group 25–44. Nearly (13.1 %, 16) of intentional injuries were among females in the age group 25–44. (See Table 3) Table 4 shows the distribution of mechanism of injury by age group and sex. Road traffic crashes were most common among males in the age-group 25–44 (35.5 %, 120) followed by 45–64 (18.0 %, 61). Fall injury was most common among males in the age-group 15–24 (24.7 %, 20) closely followed by males in 25–44 (23.5, 19) and 0–14 years (18.5 %, 15). (See Table 4) The proportion of intentional injuries/violence were significantly higher among females as compared to males. Cut wounds were significantly higher among males than females (See Table 5). Self harm was more common in the age group of 15 – 24 years where as other intents of injury were common in the age group of 25 – 44 years. Falls were significantly higher in the age of 45 years and above as compared to other nature of injuries. Sports related injuries were common among ages less than 24 years whereas in other age groups injuries occurred either during work or during travel. (See Table 6) Seasonal distribution of cases showed two peaks in the months of February and August (Fig. 1).

**Table 2:** Injuries by nature of injury and intent among patients attending the emergency of a Secondary Level Hospital in District Fatehgarh Sahib, Punjab, India, 2012

Intent of Injury	Unintentional	Intentional	Self-harm	Others	Unknown	Total
Nature of injury	No. (%)	No. (%)	No. (%)	No. (%)	No. (%)	No. (%)
Incised wound	234 (42.9)	79 (62.7)	8 (47.1)	1 (25)	4 (12.5)	326 (45)
Contusion/abrasion	128 (23.4)	20 (15.9)	5 (29.4)	1 (250	2 (6.3)	156 (21.5)
Fracture	86 (15.8)	8 (6.3)	2 (11.8)	0	16 (50)	113 (15.6)
Concussion	30 (5.5)	3 (2.4)	0 (0)	0	3 (9.4)	36 (5.0)
Laceration	29 (5.3)	2 (1.6)	0 (0)	1 (25)	3 (9.4)	35 (4.8)
Burn	11 (2.0)	0 (0)	1 (5.9)	0	0 (0)	12 (1.7)
Others	7 (1.3)	4 (3.2)	1 (5.9)	0	0 (0)	11 (1.5)
Unknown	21 (3.8)	10 (7.9)	0 (0)	1 (25)	4 (12.5)	36 (5.0)
Total	546 (100)	126 (100)	17 (100)	4 (100)	32 (100)	725 (100)

**Table 3:** Injuries by age-group, sex and intent among patients attending the emergency of a Secondary Level Hospital in District Fatehgarh Sahib, Punjab, India, 2012

Age and sex	0–14		15–24		25–44		45–64		>64		Unknown		Total		Overall
Intent	M	F	M	F	M	F	M	F	M	F	M	F	M	F	
Unintentional	35 (7)	14 (3)	113 (23)	16 (3)	164 (33)	31 (6)	79 (16)	24 (5)	13 (3)	4 (1)	0 (0)	0 (0)	404 (82)	89 (18)	493
Intentional	0 (0)	0 (0)	30 (25)	7 (6)	37 (30)	16 (13)	22 (18)	7 (6)	0 (0)	3 (2.5)	0 (0)	0 (0)	89 (73)	33 (27)	122
Self-harm	1 (8)	0 (0)	5 (42)	1 (8)	1 (8)	0 (0)	3 (25)	0 (0)	1 (8)	0 (0)	0 (0)	0 (0)	11 (92)	1 (8)	12
Other	0 (0)	0 (0)	1 (25)	1 (25)	1 (25)	0 (0)	0 (0)	1 (25)	0 (0)	0 (0)	0 (0)	0 (0)	2 (50)	2 (50)	4
Unknown	2 (11)	1 (6)	2 (11)	0 (0)	2 (11)	2 (11)	2 (11)	2 (11)	1 (6)	0 (0)	2 (11)	2 (11)	11 (61)	7 (39)	18
Total	38 (6)	15 (2)	151 (23)	25 (4)	205 (32)	49 (8)	106 (16)	34 (5)	15 (2)	7 (1)	2 (0)	2 (0)	517 (80)	132 (20)	649

**Table 4:** Injuries by age-group, sex and mechanism of injury among patients attending the emergency of a Secondary Level Hospital in District Fatehgarh Sahib, Punjab, India, 2012

Age & Sex	0–14		15–24		25–44		45–64		>64		Unknown		Total		Total
	M	F	M	F	M	F	M	F	M	F	M	F	M	F	
Road Traffic Crash	14 (4.2)	8 (2.4)	65 (19.2)	10 (3.0)	120 (35.5)	23 (7.1)	61 (18.0)	19 (5.6)	12 (3.6)	4 (1.2)	0 (0.0)	2 (0.6)	272 (80.5)	66 (19.5)	338 (100)
Quarrel	0 (0.0)	0 (0.0)	27 (24.6)	6 (5.4)	32 (29.1)	16 (14.5)	20 (18.2)	6 (5.5)	0 (0.0)	3 (2.7)	0(0.0)	0 (0.0)	79 (71.8)	31 (28.2)	110 (100)
Fall	15 (18.5)	6 (7.4)	20 (24.7)	2 (2.5)	19 (23.5)	4 (4.9)	8 (9.9)	6 (7.4)	0 (0.0)	0 (0.0)	0 (0.0)	0 (0.0)	62 (77.5)	18 (22.5)	80 (100)
Stab/cut	4 (11.1)	1 (2.8)	17 (47.2)	2 (5.6)	6 (16.7)	1 (2.8)	4 (11.1)	0 (0.0)	1 (2.8)	0 (0.0)	0 (0.0)	0 (0.0)	32 (88.9)	4 (11.1)	36 (100)
Other blunt force	3 (13.6)	0 (0.0)	8 (36.4)	0 (0.0)	6 (27.3)	0 (0.0)	3 (13.6)	1 (4.5)	1 (4.5)	0 (0.0)	0 (0.0)	0 (0.0)	21 (95.5)	1 (4.5)	22 (100)
Animal bite	1 (5.0)	0 (0.0)	3 (15.0)	1 (5.0)	8 (40.0)	3 (15.0)	2 (10.0)	2 (10.0)	0 (0.0)	0 (0.0)	0 (0.0)	0 (0.0)	14 (0.70)	6 (0.30)	20 (100)
Fire/Heat	0 (0.0)	0 (0.0)	1 (14.3)	0 (0.0)	3 (42.9)	2 (28.6)	1 (14.3)	0 (0.0)	0 (0.0)	0 (0.0)	0 (0.0)	0 (0.0)	5 (71.4)	2 (28.6)	7 (100)
Poisoning	0 (0.0)	0 (0.0)	3 (60.0)	0 (0.0)	1 (20.0)	0 (0.0)	1 (20.0)	0 (0.0)	0 (0.0)	0 (0.0)	0 (0.0)	0 (0.0)	5 (100.0)	0 (0.0)	5 (100)
Drowning	0 (0.0)	0 (0.0)	0 (0.0)	0 (0.0)	1 (100.0)	0 (0.0)	0 (0.0)	0 (0.0)	0 (0.0)	0 (0.0)	0 (0.0)	0 (0.0)	1 (100.0)	0 (0.0)	1 (100)
Gun shot	1 (100)	0 (0.0)	0 (0.0)	0 (0.0)	0 (0.0)	0 (0.0)	0 (0.0)	0 (0.0)	0 (0.0)	0 (0.0)	0 (0.0)	0 (0.0)	1 (100.0)	0 (0.0)	1 (100)
Other	0 (0.0)	0 (0.0)	3 (20.0)	3 (20.0)	4 (26.7)	0 (0.0)	4 (26.7)	0 (0.0)	1 (6.7)	0 (0.0)	2 (11.8)	0 (0.0)	14 (82.3)	3 (17.7)	17 (100)
Unknown	0 (0.0)	0 (0.0)	4 (33.3)	1 (8.3)	5 (41.7)	0 (0.0)	2 (16.7)	0 (0.0)	0 (0.0)	0 (0.0)	0 (0.0)	0 (0.0)	11 (91.7)	1 (8.3)	12 (100)
Total	38 (5.8)	15 (2.3)	151 (23.3)	25 (3.8)	205 (31.6)	49 (7.6)	106 (16.3)	34 (5.2)	15 (2.3)	7 (1.1)	2 (0.3)	2 (0.3)	517 (79.7)	132 (20.3)	649 (100)

**Table 5:** Association of intent, nature, activity during injury and mechanism of injury with sex among patients attending the emergency of a Secondary Level Hospital in District Fatehgarh Sahib, Punjab, India, 2012

Type of injury	Male *n* (%)	Female *n* (%)	Total *N*	*P*-value
Intent of injury				
Unintentional	403 (82)	89 (18)	493	0.001
Intentional	89 (73)	33 (27)	122	
Self-harm	11 (92)	1 (8)	12	
Nature of injury				
Fracture	51 (81)	12 (19)	63	0.03
Cut wound	259 (84)	51 (16)	310	
Concussion	14 (67)	7 (33)	21	
Others	135 (74)	47 (26)	182	
Activity during injury				
Work	111 (74)	39 (26)	150	0.14
Travelling	266 (81)	64 (19)	330	
Sports	17 (94)	1 (6)	18	
Others	33 (81)	8 (19)	41	
Mechanism of injury				
Road Traffic Crash	274 (81)	64 (19)	338	0.06
Fall	62 (78)	18 (22)	80	
Quarrel	79 (72)	31 (28)	110	
Others	94 (86)	16 (14)	110

**Table 6:** Association of intent, nature, activity during injury and mechanism of injury with age among patients attending the emergency of a Secondary Level Hospital in District Fatehgarh Sahib, Punjab, India, 2012

Type of injury	0–14 *N* (%)	15–24 *N* (%)	25–44 *N* (%)	45–59 *N* (%)	> = 60 *N* (%)	Total *N*	*P*-value
Intent of injury							
Unintentional	49 (10) (10.0)	129 (26) (26.2)	195 (40)	88 (18) (17.9)	31 (6) (6.3)	492 (100)	0.03
Intentional	0 (0)	37 (30)	53 (44)	23 (19) (18.8)	9 (7)	122 (100)	
Self-harm	1(8)	6 (50)	1(8)	3 (25)	1(8)	12 (100)	
Nature of injury							
Fracture	4 (7)	12 (19))	20 (32)	22 (35)	4 (7)	62 (100)	0.03
Cut wound	28 (9)	91 (29)	119 (39)	51(17)	20 (6) (6.5)	309 (100)	
Concussion	0 (0)	06 (29)	09 (42)	4 (19)	2 (10)	21 (100)	
Others	19 (10) (10.4)	49 (27)	73 (40)	28 (15)	14 (8) (7.6)	183 (100)	
Activity during injury							
Work	2 (1)	45 (30)	63 (42)	30 (20)	10 (7) (6.79)	150 (100)	<0.001
Travelling	22 (7)	73 (22)	144 (44)	67 (20)	23 (7) (7.0)	329 (100)	
Sports	10 (56) (55.6)	7 (39)	01(5)	0 (0)	0 (0)	18 (100)	
Others	9 (22)	20 (49)	04 (10)	5 (12)	3 (7)	41 (100)	
Mechanism of injury							
Road Traffic Crash	22 (7)	75 (22)	143 (43)	68 (20)	28 (8)	336 (100)	<0.001
Fall	21 (26) (25.9)	22 (27)	24 (30)	14 (17)	0 (0)	80 (100)	
Quarrel	0 (0)	33 (30)	48 (44)	21 (19)	8 (7)	110 (100)	
Others	10 (8)	46 (39)	40 (34)	16 (13)	7 (6)	119 (100)

## Discussion

This is probably the first study in India which looked at the profile of injuries admitted to the emergency department of a district level hospital and feasibility of setting up of an injury surveillance system at a secondary level for integration into the routine surveillance system. As against retrospective record based studies in the literature, this study was the result of a prospective injury surveillance project. Thus considerable attention could be paid towards collection of good quality data through repeated trainings and discussions with the Emergency Medical Officers (EMOs) who were filling the forms in spite of their busy schedules. These trainings or discussions were held when one of the authors used to go to the emergency department weekly for collection of filled forms. Considering the fact that around 1548 patients visited the emergency department in the year of the study (daily load of around 5–7), districts hospitals are not heavy load emergency settings thereby ruling out the factor of time constraint.

Males were predominantly involved in injuries similar to previous studies in different settings (Yamuragiye et al. 2013; Santikarn et al. 1999; Arscott-Mills et al. 2002; Uthkarsh et al. 2012; Chinh et al. 2007). A systematic review of injury surveillance systems in China reported 12 studies all of whom showed male preponderance ranging fom 1.9:1 to 3.1:1 (Fitzharris et al. 2011). Majority of the injuries belonged to the age group 25–44 followed by 45–64 similar to several other studies (Fitzharris et al. 2011; Deng et al. 2010). This preponderance for adult males could be attributed to the fact that males in this age group tend to be more involved in outdoor occupations with wide ranging travel needs and other high risk activities (Holder et al. 2001).

Seasonal variation with a bimodal peak was observed with maximum number of injuries and crashes reported during the months of February and August. This might be probably due to foggy conditions in the month of February and slippery roads due to rains during August. However, some evidence in the literature suggests that injuries peak during the summer months of June-August (Foltran et al. 2013; Morrison et al. 1999). There is a sudden dip in the cases in the months of May to July. This may be due to underreporting because of lack of supervision due to non availability of staff during this period. Sustained motivation and monitoring will improve the data collection process by the system. Political commitment in the form of integration along with the existing surveillance system is essential for sustainability.

More than half of the patients admitted to an emergency department of a hospital sustained road traffic injuries similar to previous studies in low and middle income countries countries (Yamuragiye et al. 2013; Uthkarsh et al. 2012; Chinh et al. 2007; Zavala et al. 2008; John et al. 2008). However few studies have reported fall injury as the most common mechanism of injury (Musharrafieh et al. 2011; Cardona et al. 2008). Worldwide road traffic injuries and falls are among the top leading causes of injuries in different settings. Road traffic injuries were mostly young adult males similar to most other studies (Arscott-Mills et al. 2002; Uthkarsh et al. 2012; John et al. 2008; Cardona et al. 2008; Mtonga & Zavala 2008; Ganveer & Tiwari 2005; Dandona et al. 2006; Jirojwong et al. 2002; Singh & Goel 2011). Several studies have reported that two-wheelers are most vulnerable to crashes similar to the findings in the present study (Santikarn et al. 1999; Uthkarsh et al. 2012; Chinh et al. 2007; Cardona et al. 2008; Jirojwong et al. 2002; Singh & Goel 2011).

Fall injuries were common in the younger age group of 25–44 mostly related to work or travel followed by 0–14 and predominantly among males similar to another study by Grivna et al (Grivna et al. 2014). On the contrary, previous studies have shown that falls were a significant cause of both death and injury in women and older adults in high income countries and in India as well (Cardona et al. 2008). This difference in age distribution of falls might be due to the poor health seeking behaviour of elderly and tendency to seek medical care only when the condition becomes severe (WHO 2007). Also most of the falls in the eldery age group do not result in serious injuries (Tripathy et al. 2015).

In the present study, majority of the falls occurred at home as against workplace in other studies (Cardona et al. 2008; Grivna et al. 2014; Tuma et al. 2013; Ng et al. 2010). The high rates of falls in younger males at home indicate the high level of risk encountered by men and older children in rural India as they go about their usual household and playful activites. However, we need to carry out further studies to explore this in further details.

Among the patients who were referred, nearly 76 % of them had encountered a road traffic crash. About 66 % of the patients referred had injuries on head and face. Also five out of six deaths were as a consequence of road traffic injuries. Thus road traffic injuries account for majority of severe injuries thereby demanding immediate attention. Thus, the feasibility of establishing well equipped acute head injury units and a responsive referral system at the district level needs to be researched upon.

Around 25 % of patients who had sustained road traffic injuries were under the influence of alcohol. Nearly 45 % of such crashes were reported during the weekends similar to other studies whereas some studies also reported more crashes during weekdays (Ricci et al. 2008; Híjar et al. 2000). Thus strict penalties should be imposed on the offenders and at the same time public should be made aware of the risk of crashes in case of drinking while driving. Around 90 % of those who sustained road traffic injuries while riding a vehicle did not wear a helmet or seat belt. Four out five deaths due to road traffic crash had head and neck injuries which could have been avoided by the use of helmets. Among those who did not wear a helmet, 42 % (92) had head injuries while 25 % (54) suffered injuries to the face. Also, among those who wore a helmet, around 30 % received head injuries which probably suggests overreporting of helmet use due to soial desirability bias or use of helmets which do not conform to the safety standards. This mandates strict legislative action and awareness activities. With such a large share of road traffic crashes among injury cases and especially among severe injury cases, there is an urgent need to put injury prevention policy at the forefront rather than divesting large volume of resources towards acute trauma care.

Lack of reliable information on injury deaths, disabilities and hospitalizations puts injury quite low on the national public health agenda which leads to poor funding opportunities and scarcity of resources. A robust system of routine injury surveillance is essential to generate data for effective decision making. Injury surveillance can identify, monitor and prevent injuries and their risk factors (Holder et al. 2001; Forjuoh & Gyebi-Ofosu 1993; Halperin 1996). There are other examples of emergency based surveillance systems such as the Weapon Related Injury Surveillance System (WRISS) in the United States (Barber et al. 1998) which was implemented to track firearm related injuries. The Violence Related Injury Surveillance System (VRISS), Australian National Injury Surveillance and Prevention Program (NISPP) and the Canadian Hospital Injury Reporting and Prevention Program (CHIRPP) have also used emergency department data to describe the profile of injuries (Ward et al. 2002; Australian National Injury Surveillance Unit (NISU); MacKenzie & Pless 1999). National injury surveillance systems have also been initiated in Asian countries like Thailand and China. Sentinel surveillance system in China covers 10 % of the population. The sentinel data have been used to estimate overall injury burden, identify leading risk factors and high-risk groups (Li & Baker 1991). Thailand also initiated a provincial injury surveillance system with reportingfrom five large hospitals in Bangkok and four regions of the country. The system reported coverage of 98.8 %, whereas completeness and reliability of recording ranged from 80.6–100 % (Santikarn et al. 1999). In another similar resource constraint setting in the Sub-saharan Africa, a trauma registry and injury severity measurement were found to be feasible and useful to assess the effectiveness and efficiency of acute care for the injured (Kobusingye & Lett 2000).

In this study, the data on the actual number of patients with injuries admitted to the emergency department could not be obtained. However, the surveillance system could capture almost all the medico-legal cases although the proportion of such cases captured by the surveillance system could not be ascertained. There might also be cases of injuries which were not registered as medico-legal cases. These are the cases with injuries not considered to have any medico-legal implications by the physician based on the circumstances that led to the injury such as injuries sustained due to a fall at home caused by a slippery surface. Thus the decision to register a medico-legal is with the treating physician. If there is any suspicion regarding the causation of the injury, he might call the police and register a medico-legal case.

Because emergency department surveillance generates large numbers of injury records in a short period of time, the cost per injury of collecting data at emergency departments is lower than for a population based survey (Mulder 1997). The surveillance system was able to capture injury related information in a simple one-page proforma which could be administered easily without hampering the routine schedule of the emergency department medical staff. A total of 4 medical officers were trained on the data collection proforma. They themselves trained other medical officers who were posted in rotation at the emergency department. In this pilot study at a district level hospital, around 14 injury cases reported to the emergency department in 1 week i.e. 2 cases per day. The nursing staff in the emergency department could be trained to fill the proforma which might hardly take 10 min for each. The data entry operator under IDSP at the district level could be used to enter the data which will hardly consume less than 30 min in a week. The district epidemiologist at the district level could be the nodal person overseeing the surveillance system. Thus, with the existing resources, we can establish a sustainable injury surveillance system at the district hospital level which will provide us with a large database on injury burden and characteristics in the country for effective decision making.

The missing values in various key variables suggest the need for better training and a comprehensive manual for those collecting data. The training and the manual should in detail highlight the significance of each variable collected. Variables such as time of injury, mode of transportation to the hospital, outcome of injury, alcohol use and substance use which had higher proportion of missing values need particular attention during training.

A major limitation of a hospital based surveillance is that the patients who reported an injury at the hospital are just a tip of the iceberg with fatal (on site of injury) cases missed and also substantial number of injury cases who do not avail health care services. Collecting injury related information from patients with severe injuries requiring immediate referral was a challenge because of time limitations as these were time critical. In such cases, important injury related information can be built in the referral form for quick capture. The injuries resulting in immediate death also might not have been reported to the hospital such as suicides due to hanging, drowning or road traffic crashes resulting in death at the place of the event itself. This will result in underestimation of injury burden. The research revealed limitations in record keeping of injury patients in the emergency department of a hospital. Although the EMOs were trained to fill the injury proforma but frequent change of emergency department staff and lack of motivation on the part of the EMOs were some of the problems encountered that suggests implications for the nurses working in the emergency department. There is high turnover of health personnel particularly doctors in the emergency department which hampers ongoing data collection. This issue was addressed through regular monitoring and periodic visits by the investigators wherein new medical officers were sensitized and trained about the surveillance system. However there is a need to incorporate injury surveillance as a routine feature in the emergency department.

## Conclusion

Addressing the rising burden of injuries with competing healthy priorities and without robust data on injury burden and risk factors, is a major challenge for developing countries. There is need for various sectors like health, police, transport to come together and develop multifactorial intervention for injury prevention. Developing better and sustainable systems of routine injury surveillance or trauma registries is essential to generate reliable information for formulating effective intervention policies.

## Abbreviations

CHC: Community health centre
ED: Emergency Department
EMP: Emergency medical officer
MLCs: Medico legal cases
PHC: Primary health care
